# Supplementary material for: Spinal cord lesions in relapsing-remitting multiple sclerosis: Prognostic value of intrathecal production of immunoglobulins
Source: PLoS One. 2025 Dec 5;20(12):e0337515. doi: 10.1371/journal.pone.0337515 (PMC12680195; doi:10.1371/journal.pone.0337515)
Supplement: Attachment — Submitted filename: RebuttalLetter_081125.docx [file pone.0337515.s003.docx]

Dear editor,

We would like to thank the reviewers for their replies and additional comments. The comments are addressed below:

**Review #1:** I would suggest removing the following sentence:
“With respect to the spinal cord, there is no conclusive evidence of a difference in lesion detection between 1.5T and 3T MRI systems.”
Please present number of scans at each magnetic field instead. Please see manuscript: "Impact of 3 Tesla MRI on interobserver agreement in clinically isolated syndrome: A MAGNIMS multicentre study."

**Authors**: We appreciate the reviewer’s suggestion and highlighting the MAGNIMS multicentre study. We agree that 3T systems can improve signal-to-noise and, as reported in the mentioned paper, may yield higher interobserver agreement for spinal cord lesions. However, that study also notes that it has not conclusively been demonstrated that 3T achieves higher lesion detection than lower field strengths in spinal cord MRI. Our sentence was intentionally phrased to reflect this nuance.

Reporting the number of scans acquired at 1.5T versus 3T in our dataset would not address the reviewer’s core concern. Field-strength counts primarily reflect practice patterns and site-level availability, not diagnostic yield. Our study was neither designed nor powered to perform a head-to-head test of lesion detection across field strengths, and presenting raw counts could be misinterpreted as evidence for, or against, superiority.

For these reasons, we respectfully propose to retain the sentence. To acknowledge the reviewer’s point and improve clarity, we revised the surrounding text to make the distinction explicit and added a reference to the study mentioned (lines 113-116 in clean version): “*For the spinal cord, while 3T may improve interobserver agreement [14], there is currently no conclusive evidence that 3T increases lesion detection relative to 1.5T. Consequently, scans obtained at either field strength were considered eligible for inclusion [15, 16].*”

**Review #1:** Please add a sentence in the methods to specify that patients were DMT-naïve at the time of CSF testing.

**Authors**: We thank the reviewer for this suggestion. We have added the following sentence to the Methods (lines 90-91):

“*All patients were disease-modifying therapy (DMT)–naïve at the time of CSF sampling*.”

**Review #1:** Table 1: There is mention of IgM twice. The first should be IgG.

**Authors**: We appreciate the careful reading. This has been corrected from IgM to IgG in Table 1. We have reviewed the table, caption, and corresponding in-text references to ensure consistency throughout.

**Reviewer #2:** It seems that the authors did not address the point: “As acknowledged by the authors, disease-modifying therapies (DMTs) represent a potential confounder that is not sufficiently addressed in the analyses.”

**Authors:** We thank the reviewer for raising this important issue and apologize for not addressing it adequately in our previous reply. For the longitudinal analyses we adjusted for DMT exposure by including DMT category as a covariate in the time-to-event models (we clarified the explanation a bit in lines 141-143 in the clean version). Given the available data, sample size, and the retrospective study design, we believe this represents the most robust adjustment feasible with our dataset. We fully agree, however, that residual confounding cannot be completely eliminated in nonrandomized, retrospective data. We therefore explicitly acknowledge this limitation in the revised text in the limitations section (lines 286 – 290 in the clean version). Importantly, this concern does not affect the baseline analyses, as all patients were DMT-naïve at baseline.

We hope these clarifications address the reviewer’s concern while accurately reflecting the strengths and constraints of our dataset.

**Reviewer #2:** In response to the comment “Given the distribution of lesion count, multivariate linear regression may not be the most appropriate modeling approach,” the authors state that “As lesion count is the dependent variable of the model here, the distribution of this variable is not of relevance to the assumptions of the regression model.” This statement appears inaccurate, as the distribution of the dependent variable is crucial for ensuring that model assumptions are met. Depending on its distribution, a count regression model (e.g. Poisson or negative binomial) may be more appropriate.

**Authors:** We thank the reviewer for this important correction and apologize for our imprecise statement. Accordingly, we have re-estimated the lesion-count models using count regression. Because the data were overdispersed, we employed a negative binomial model (using glm.nb in R) rather than Poisson. The Methods section has been amended to describe the modelling approach (lines 130-133 in the clean version), and the Results have been updated accordingly (lines 180-181 in the clean version). These changes produced minor numerical differences but did not alter the substantive conclusions of the analysis. We appreciate the reviewer’s guidance, which has improved the statistical rigor and clarity of the manuscript.

**Reviewer #2:** According to the authors’ response, the point “While a relatively large number of participants contributed to the cross-sectional analysis, a major limitation of the study is the substantial drop-out in the longitudinal analyses” also seems not to have been addressed.

**Authors:** We thank the reviewer for highlighting this point and apologize for not addressing it sufficiently in our earlier reply. To clarify, the issue is not loss to clinical follow-up, but lack of follow-up spinal cord MRI. Serial brain MRI is routine, while spinal cord MRI is not. Therefore, some participants had no follow-up cord imaging despite ongoing clinical follow-up. This expected pattern explains the smaller longitudinal imaging cohort. These limitations are now explicitly discussed in the Limitations section (lines 278-282 in the clean version), where we have expanded the explanation to address the reviewer’s point.

***Earlier rebuttal letter (first round over revisions)***

Dear editor,

We would like to thank the reviewers for their thorough review of the manuscript and the comments. We believe that the manuscript improved because of it. Below we discuss all editorial and reviewer comments and the changes made to the manuscript.

*Editorial comments*

*1 Please ensure that your manuscript meets PLOS ONE's style requirements, including those for file naming.*

**Authors**: We walked through the requirements and made the necessary changes to the manuscript formatting.

*2 Please note that funding information should not appear in any section or other areas of your manuscript. We will only publish funding information present in the Funding Statement section of the online submission form. Please remove any funding-related text from the manuscript.*

**Authors**: This has now been removed

*3 When completing the data availability statement of the submission form, you indicated that you will make your data available on acceptance. We strongly recommend all authors decide on a data sharing plan before acceptance, as the process can be lengthy and hold up publication timelines. Please note that, though access restrictions are acceptable now, your entire data will need to be made freely accessible if your manuscript is accepted for publication. This policy applies to all data except where public deposition would breach compliance with the protocol approved by your research ethics board. If you are unable to adhere to our open data policy, please kindly revise your statement to explain your reasoning and we will seek the editor's input on an exemption. Please be assured that, once you have provided your new statement, the assessment of your exemption will not hold up the peer review process.*

**Authors**: an anonymized dataset containing all data necessary to repeat/reproduce the analyses has already been deposited in the Open Science Foundation repository mentioned in the manuscript. At the moment of acceptance, this repository will be made public.

*4 Thank you for stating the following in the Competing Interests section: “I have read the journal's policy and the authors of this manuscript have the following competing interests: Niels Franken has nothing to disclose. Daniel Kreiter, Demmie Bouweriks, Stephanie Knippenberg, Oliver Gerlach, Raymond Hupperts received institutional research grants and a grant from Nationaal MS fonds not related to this research; RH received fees for lectures and advisory boards from Biogen, Merck and Genzyme-Sanofi.” We note that you received funding from a commercial source: Nationaal MS fonds. Please provide an amended Competing Interests Statement that explicitly states this commercial funder, along with any other relevant declarations relating to employment, consultancy, patents, products in development, marketed products, etc.*

*Within this Competing Interests Statement, please confirm that this does not alter your adherence to all PLOS ONE policies on sharing data and materials by including the following statement: "This does not alter our adherence to PLOS ONE policies on sharing data and materials.” (as detailed online in our guide for authors* [*http://journals.plos.org/plosone/s/competing-interests*](http://journals.plos.org/plosone/s/competing-interests)*).  If there are restrictions on sharing of data and/or materials, please state these. Please note that we cannot proceed with consideration of your article until this information has been declared.*

**Authors**: Nationaal MS fonds (the Dutch MS foundation) is not a commercial organization, this is a non-profit foundation. The grant received from this foundation is also not related to this study but for another study.

*5 Please include captions for your Supporting Information files at the end of your manuscript, and update any in-text citations to match accordingly.*

**Authors**: This manuscript has no supplements

*Reviewer comments*

*Reviewer #1: Please provide further explanation why progressive MS patients were excluded.*

**Authors**: Only relapse-onset patients and not progressive-onset were selected as the baseline moment can be better determined for relapse-onset patient and to achieve a more homogeneous group. The explanation has been added to the Methods section as well (lines 86-88).

*Reviewer #1: Please include MRI protocols including sequences obtained, magnet strength, etc.*

**Authors**: In the Methods-section we added a section giving an explanation on the imaging protocols and scanners. Additionally, a section to the Limitations-section was added to explain the limitation this causes. (lines 105-115)

*Reviewer #1: Please include normal cut-off values for CSF IgM and IgG indices.*

**Authors**: The relevant cut-off value used is already mentioned in the Methods-section, when using the Reiber formula, the cut-off is above 0% to consider the value of the CSF IgG and IgM as high correcting for blood-brain barrier permeability and serum IgG and IgM. For a detailed description of this method see Reiber, 1998. We noticed the reference to the Reiber papers to be missing in the methods section, these have now been added. The mentioned cut-off is the cut-off used in other work as well (e.g. Oechtering 2021 and 2022). Nevertheless, we expanded the explanation a bit, to give a global idea of what the goal of using the Reiber formula is (lines 122-126).

In the baseline characteristics table (Table 1) we now added often used cut-offs for the mentioned IgG and IgM index in the table.

*Reviewer #1: I don't believe impact of DMT is adequately addressed in the current study. Evaluating the first DMT used (vs. highest efficacy DMT used) would make more sense for the current study. Further, would it be possible to compare impact of DMT efficacy using propensity score matching?*

**Authors:** With the reviewers suggestion we decided to add a sensitivity analysis using first instead of highest treatment category. This did not result in a relevant difference in the results. We added this to the main text, see linesn 208-210. In regard to the comment whether it would be possible to compare impact of DMT categories on cord lesions using propensity score matching, that is not the goal of this study as we are not comparing interventions but looking into a baseline predictor (for the DMT effect question analyzed on this database using propensity score matching, see Kreiter et al, 2023).

*Reviewer #1: Would it be possible to provide data on lesion volumes in addition to lesion counts?*

**Authors:**  This would certainly be interesting, but unfortunately this is not possible from current data, as the performed clinical spinal cord MRI protocols did not include 3D sequences to be able to determine lesion volumes.

*Reviewer #1:* Impact of DMT on IgG and IgM index warrants further discussion. Could this impact the prognostic value of these biomarkers if they are considered dynamic and DMT dependent?

IgG and IgM indices were determined using CSF at diagnosis (so before start of treatment), which means there is no impact of DMTs there (this is already mentioned in the manuscript, lines 158-159). However, in some cases patients could have been treated with corticosteroids shortly before CSF acquisition, which can impact these indices. Therefore, these patients were excluded (n=21, see figure 1 in the manuscript).

*Reviewer #1: Methods: It remains unclear exactly how recurrent events were analyzed in this model. Were adjustments such Andersen–Gill model used or some other methods? Were there any violations in the assumptions of the CPH model?*

**Authors:** The reviewer is right that in fact the model applied here is an Andersen-Gill model, as the analysis uses coxph(Surv(start, stop, event) ~ X1 + …, id=PATIENT_ID, data = data) command in R to allow multiple events per subject and account for within-subject correlation. We corrected the terminology used in the Methods-section (lines 135-137).

There were no violations of the assumptions of the model. Schoenfeld’s residual plots were used to assess the proportional hazards assumption and the variance inflation factor to check for collinearity. A mention that this has been checked has now been added to the manuscript (lines 137-138).

*Reviewer #1: Abstract: remove “more” in the line “… identify patients prone to more future …”*

**Authors:** Thank you for the suggestion, this has been removed from that sentence.

*Reviewer #1: Abstract: prefer inclusion of number included participants and baseline demographics if possible within word/character count limits*

**Authors:** We added a sentence with inclusion numbers to the Results-part of the Abstract.

*Reviewer #2: In the longitudinal evaluation, it is important to specify which spinal cord regions were covered by MRI. Was there variability in spinal cord coverage across patients?*

**Authors:** Yes there was variation, the thoracic cord was not always included, and this is mentioned in the manuscript, “65% of spinal cord MRIs in this subset also included the complete thoracic cord” (lines 157-158).

*Reviewer #2: The potential use of heterogeneous spinal cord MRI protocols across follow-up scans might be a relevant confounding factor.*

This is true and this is now more extensively discussed in the Limitations-section (lines …)

*Reviewer #2: The retrospective derivation of spinal cord lesion burden from radiology reports is a major limitation. In how many cases were MRI images re-evaluated by the authors?*

**Authors:** All spinal cord MRI reports and images were checked by one of the investigators with experience with spinal cord imaging (DK), when the database was constructed (also before adding clinical / CSF data). In case of conflicts with the original report, a third rater was consulted (OG). This has now been better explained within the text of the manuscript (lines 282-286).

*Reviewer #2: Given the distribution of lesion count, multivariate linear regression may not be the most appropriate modeling approach.*

**Authors:** As lesion count is the dependent variable of the model here, the distribution of this variable is not of relevance to the assumptions of the regression model.

*Reviewer #2: Cox proportional hazards models estimate hazard ratios, not risk ratios as stated in the manuscript, and this terminology should be corrected.*

**Authors:** Thank you, this is correct and has now been corrected in the text.

*Reviewer #2: The statement “66 patients had a total of 118 follow-up spinal cord MRIs” corresponds to fewer than two MRIs per patient on average, which seems low and requires clarification.*

**Authors**: It concerns 118 *follow-up* MRIs (so in addition to the baseline cord MRI), this is a bit fewer than 2 follow-up cord MRIs per patient, but given the median follow-up time of 2 years this is not that low.

While a relatively large number of participants contributed to the cross-sectional analysis, a major limitation of the study is the substantial drop-out in the longitudinal analyses.

*Reviewer #2: In the abstract, correct “IgF” to “IgG.”*

**Authors**: This has been corrected in the new version of the manuscript.

*Reviewer #2: The abstract should indicate the total number of patients included, as well as details on follow-up duration.*

**Authors**: This is now added to the abstract (lines 29-30 & 35-36).
